# Michigan State Dental Society

**Published:** 1886-04

**Authors:** 


### Michigan State Dental Society.

The annual meeting of this body was held in Ann Arbor on the 16th to the 19th of March, inclusive. The proceedings were more varied than is usual on such occasions. The mornings of each day except the last were devoted to prosthetic dentistry, and the afternoons to operative dentistry. The members taking the place of the students of the dental college, there was an excellent opportunity for clinics, and it was largely utilized, the members seeming to enjoy the arrangements very much.

The opinion was unanimous that a clinic room of a dental college is a superior place for this part of dental society work. There was an abundance of material for clinics, and in great variety. There were many fine operations. This is fully emphasized by saying that Drs. Geo. L. Field, E. C. Moore, C. H. Harroun, J. E. Low, and C. H. Land were among the operators.

Among the papers read, that elicited great interest, was one by Dr. J. N. Martin, lecturer on oral pathology and surgery in the dental college, descriptive of an extensive abscess and necrosis of the lower jaw, embracing treatment.

A very interesting lecture on comparative odontology was delivered before the society by Prof. C. L. Ford, of the University. To those who know Prof. Ford—and who does not—nothing need be said as to the character of the lecture.

The paper of Dr. Martin and the report of the lecture will both be given in the May REGISTER.

There was at this meeting a larger attendance than ever before; there were of members and visitors about one hundred, and eighty students. The work of the society, though out of the usual course, was eminently successful; and all went away feeling that they had been greatly profited.

A synopsis of the proceedings will be given in the May REGISTER.

